# Selective PPARδ agonist seladelpar suppresses bile acid synthesis by reducing hepatocyte CYP7A1 via the fibroblast growth factor 21 signaling pathway

**DOI:** 10.1016/j.jbc.2022.102056

**Published:** 2022-05-20

**Authors:** Tetsuya Kouno, Xiao Liu, Huayi Zhao, Tatiana Kisseleva, Edward E. Cable, Bernd Schnabl

**Affiliations:** 1 Department of Medicine, University of California San Diego, La Jolla, California, USA; 2 Department of Surgery, University of California San Diego, La Jolla, California, USA; 3 CymaBay Therapeutics, Newark, California, USA; 4 Department of Medicine, VA San Diego Healthcare System, San Diego, California, USA

**Keywords:** bile acids, peroxisome proliferator–activated receptor, hepatocyte, cholesterol 7 alpha-hydroxylase, liver disease, NAFLD, FBS, fetal bovine serum, FXR, Farnesoid X receptor, JNK, c-Jun N-terminal kinase, PBC, primary biliary cholangitis, PPAR, peroxisome proliferator–activated receptor, PPARA, PPAR-alpha, PPARD, PPAR-delta, PPARG, PPAR-gamma, Shp, small heterodimer partner

## Abstract

Peroxisome proliferator–activated receptor delta (PPARδ) agonists have been shown to exert beneficial effects in liver disease and reduce total bile acid levels. The mechanism(s) whereby PPARδ agonism reduces bile acid levels are, however, unknown, and therefore the aim of the present study was to investigate the molecular pathways responsible for reducing bile acid synthesis in hepatocytes, following treatment with the selective PPARδ agonist, seladelpar. We show that administration of seladelpar to WT mice repressed the liver expression of cholesterol 7 alpha-hydroxylase (Cyp7a1), the rate-limiting enzyme for bile acid synthesis, and decreased plasma 7α-hydroxy-4-cholesten-3-one (C4), a freely diffusible metabolite downstream of Cyp7a1. In primary mouse hepatocytes, seladelpar significantly reduced the expression of *Cyp7a1* independent of the nuclear bile acid receptor, Farnesoid X receptor. In addition, seladelpar upregulated fibroblast growth factor 21 (Fgf21) in mouse liver, serum, and in cultured hepatocytes. We demonstrate that recombinant Fgf21 protein activated the c-Jun N-terminal kinase (JNK) signaling pathway and repressed *Cyp7a1* gene expression in primary hepatocytes. The suppressive effect of seladelpar on *Cyp7a1* expression was blocked by a JNK inhibitor as well as in the absence of Fgf21, indicating that Fgf21 plays an indispensable role in PPARδ-mediated downregulation of *Cyp7a1*. Finally, reduction of CYP7A1 expression by seladelpar was confirmed in primary human hepatocytes. In conclusion, we show that seladelpar reduces bile acid synthesis via an FGF21-dependent mechanism that signals at least partially through JNK to repress CYP7A1.

Disrupted bile acid metabolism is closely associated with the development of metabolic and liver diseases, including nonalcoholic fatty liver disease (1), alcohol-associated liver disease (2), primary biliary cholangitis (PBC), and primary sclerosing cholangitis (3, 4). Farnesoid X receptor (FXR) and small heterodimer partner (Shp) play a key role in regulating bile acid synthesis in the liver. Bile acid–activated FXR induces Shp which acts as a corepressor of liver X receptor alpha, hepatic nuclear factor 4 alpha, and liver receptor homolog 1 to reduce the expression of cholesterol 7 alpha-hydroxylase (Cyp7a1) (5), which catalyzes the rate-limiting step in the conversion of cholesterol to bile acids (6). Although FXR/Shp pathway plays a central role in the negative feedback regulation of bile acid synthesis, dietary feeding of bile acid to Shp-null mice results in further reduction of Cyp7a1 expression (7, 8), implying that the FXR/Shp cascade may not be the only pathway for downregulating bile acid synthesis.

Peroxisome proliferator–activated receptors (PPARs) are members of the nuclear receptor family of ligand-activated transcription factors and include PPAR-alpha (PPARA), PPAR-delta (PPARD), and PPAR-gamma (PPARG). A PPARA agonist inhibits bile acid synthesis (9) by attenuating the transcription of *Cyp7a1* (10). Our previous study showed that the selective PPARD agonist seladelpar (MBX-8025) alleviates ethanol-induced liver disease in mice by reducing the total bile acid pool and bile acid concentrations in the liver, small intestine, and systemic circulation, and changes bile acid composition (11). Seladelpar significantly decreased bile acid precursor 7 alpha-hydroxy-4-cholesten-3-one (C4) and total bile acids in patients with PBC (12, 13), indicating that PPARD reduces bile acid synthesis, in humans. The aim of our present study was to investigate the molecular mechanism(s) whereby seladelpar, a selective PPARD agonist, decreases bile acid synthesis.

## Results

### The selective PPARD agonist seladelpar reduces hepatic *Cyp7a1* expression in mice

**Figure 1:**
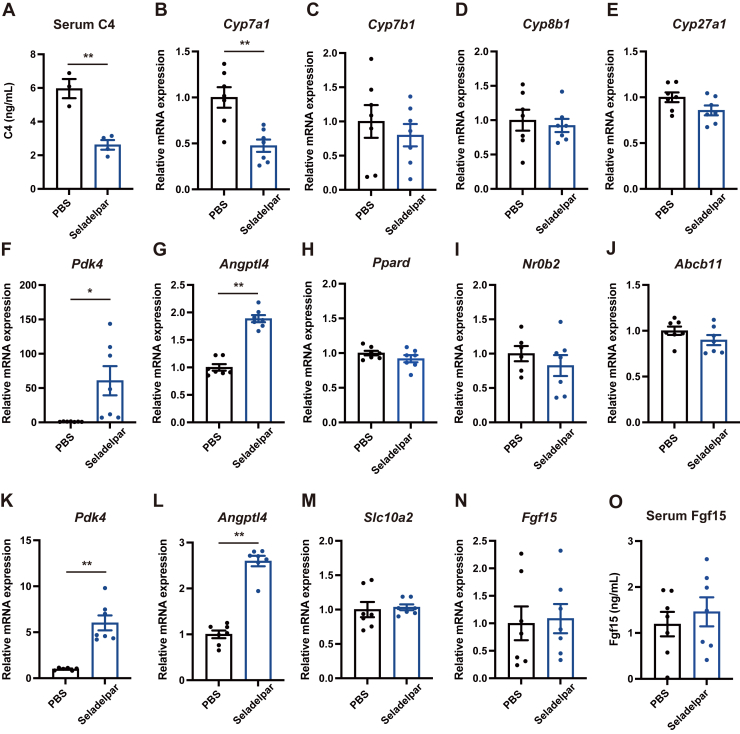
**Effect of seladelpar on gene expression *in vivo*.** Male WT C57BL/6 mice were gavaged with seladelpar (10 mg/kg body weight) and harvested after 6 h. *A*, serum C4. *B–J*, gene expression in the liver. *K–N*, gene expression in the ileum. *O*, Fgf15 in mouse serum. Results of gene expression were obtained from two technical replicates. Data are presented as mean ± S.E.M., ∗*p* < 0.05 and ∗∗*p* < 0.01 denotes the significant difference between PBS and seladelpar.

To examine the effect of the selective PPARD agonist seladelpar on bile acid homeostasis *in vivo*, the expression of genes involved in bile acid synthesis was investigated in the liver and small intestine 6 h after oral administration of seladelpar in mice. Seladelpar reduced serum 7α-hydroxy-4-cholesten-3-one (C4) (Fig. 1*A*), a marker for *de novo* bile acid synthesis, and hepatic expression of *Cyp7a1* (Fig. 1*B*), which encodes the rate-limiting enzyme for bile acid synthesis. No significant change was observed in hepatic *Cyp7b1* (Fig. 1*C*), *Cyp8b1* (Fig. 1*D*), or *Cyp27a1* (Fig. 1*E*), which are responsible for alternative pathways for bile acid synthesis. PPARD target genes pyruvate dehydrogenase kinase 4 (*Pdk4*) and angiopoietin-like 4 (*Angptl4*) were significantly induced by seladelpar in liver and terminal ileum (Fig. 1, *F*, *G*, *K*, and *L*), whereas *Ppard* expression was unchanged (Fig. 1*H*). FXR target genes *Nr0b2* (encoding Shp) or ATP binding cassette subfamily B member 11 (*Abcb11*) (known as bile salt export pump) in the liver were not significantly changed (Fig. 1, *I* and *J*), indicating that the FXR pathway is not affected by seladelpar. *Slc10a2* (also known as apical sodium-dependent bile acid transporter), which is responsible for the uptake of conjugated bile acids into enterocytes of the terminal ileum, showed no significant difference between the groups (Fig. 1*M*). Fibroblast growth factor 15 (Fgf15), which negatively regulates bile acid synthesis in hepatocytes, was unchanged following seladelpar administration (Fig. 1, *N* and *O*). These results suggest that seladelpar regulates bile acid synthesis in the liver without affecting the gut-liver FXR-Fgf15 axis and enterohepatic circulation.

### Seladelpar reduces Cyp7a1 expression in primary mouse hepatocytes

**Figure 2:**
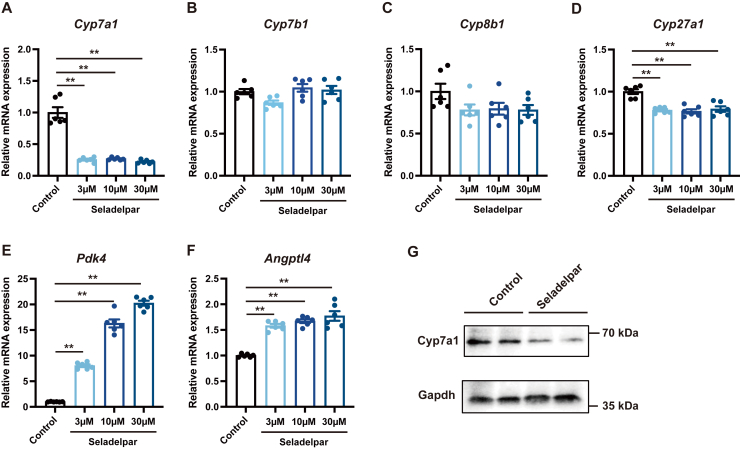
**Effect of seladelpar on gene expression in primary mouse hepatocytes.***A–F*, primary mouse hepatocytes were treated with seladelpar (3–30 μM) for 48 h, and gene expression analysis was performed. *G*, Western blot analysis of primary mouse hepatocytes treated with seladelpar (10 μM) for 72 h. qPCR data are presented as mean ± S.E.M. of at least three independent replicates. ∗∗*p* < 0.01 denotes the significant difference between control and seladelpar.

To evaluate the direct effect of seladelpar on Cyp7a1, primary hepatocytes isolated from WT C57BL/6 mice were treated with seladelpar. Seladelpar significantly reduced the expression of *Cyp7a1* (Fig. 2*A*) and slightly decreased the expression of *Cyp27a1*, whereas *Cyp7b1 and Cyp8b1* were not significantly affected (Fig. 2, *B*–*D*). PPARD target genes *Pdk4* and *Angptl4* were induced by seladelpar (Fig. 2, *E* and *F*), whereas *Ppard, Ppara,* and *Pparg* expression was unchanged (Fig. S1). Immunoblot analysis confirmed that Cyp7a1 protein was downregulated by seladelpar (Fig. 2*G*). The downregulation of *Cyp7a1* gene expression in primary mouse hepatocytes was confirmed by other PPARD agonists, REN001 and ASP0367 (Fig. S2, *A* and *B*).

### Seladelpar reduces *Cyp7a1* gene expression independent of the FXR pathway

**Figure 3:**
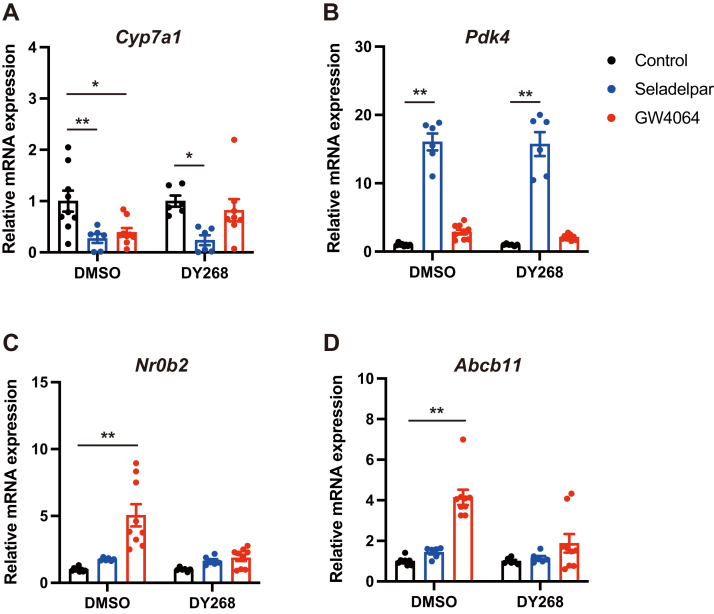
**Effect of seladelpar on the FXR pathway in primary mouse hepatocytes.** Primary mouse hepatocytes were treated with either seladelpar (10 μM) or the FXR agonist GW4064 (10 μM) in combination with either DMSO or the FXR antagonist DY268 (10 μM) for 48 h, and gene expression analysis was performed. Data are presented as mean ± S.E.M. of at least three independent replicates. ∗*p* < 0.05 and ∗∗*p* < 0.01 denote the significant difference between control and seladelpar or GW4064 in each condition. FXR, Farnesoid X receptor.

Since the FXR pathway plays an important role in the negative feedback of bile acid synthesis, we next examined whether modulation of the FXR pathway is involved in the effect of seladelpar on *Cyp7a1* expression. The FXR agonist, GW4064, repressed *Cyp7a1* gene expression (Fig. 3*A*) accompanied with a significant increase in FXR target genes, *Nr0b2* and *Abcb11* (Fig. 3, *C* and *D*), while seladelpar reduced *Cyp7a1* expression (Fig. 3*A*) without changing FXR target genes (Fig. 3, *C* and *D*). The suppressive effect of GW4064 on *Cyp7a1* expression was blocked by the treatment with the FXR antagonist, DY268 (Fig. 3*A*). On the other hand, seladelpar still decreased *Cyp7a1* expression in the presence of DY268 (Fig. 3*A*), indicating that seladelpar downregulates *Cyp7a1* gene expression independent of the FXR pathway.

### Fgf21 expression is induced by seladelpar independent of PPARA

**Figure 4:**
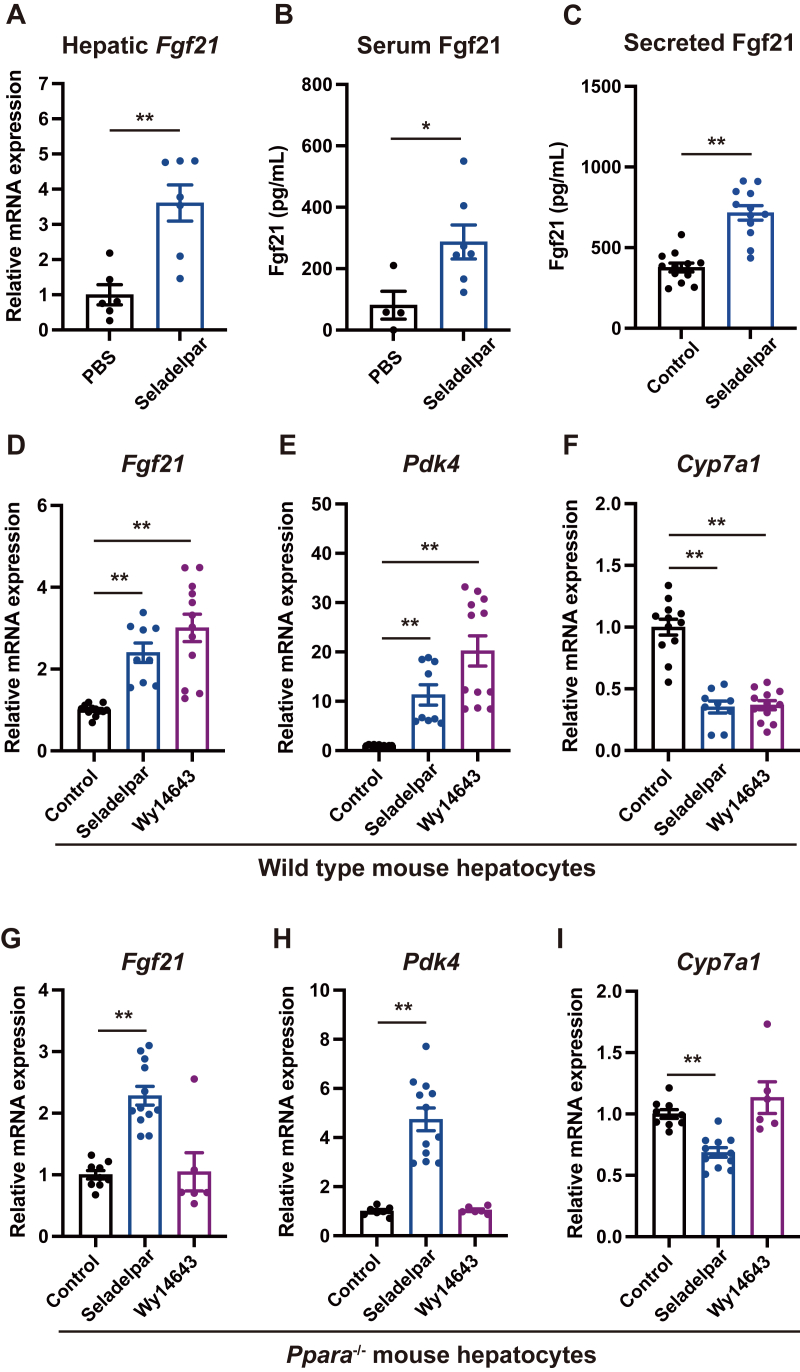
**Effect of seladelpar on Fgf21 expression.***A* and *B*, mice were orally gavaged with seladelpar (10 mg/kg body weight) and harvested 6 h later. *A*, hepatic gene expression of *Fgf21*. *B*, serum levels of Fgf21. *C*, primary mouse hepatocytes were treated with seladelpar (10 μM) for 48 h, and Fgf21 was measured in the medium. *D–F*, primary mouse hepatocytes were treated with seladelpar (10 μM) or the PPARA agonist Wy14643 (10 μM) for 48 h and mRNA expression was examined. *G–I*, primary hepatocytes isolated from *Ppara*^-/-^ mice were treated with seladelpar (10 μM) or Wy14643 (10 μM) for 48 h and mRNA expression was examined. Data are presented as mean ± S.E.M. of at least three independent replicates. ∗*p* < 0.05 and ∗∗*p* < 0.01 denote the significant difference between control and seladelpar or Wy14643. PPARA, peroxisome proliferator–activated receptor alpha.

Since Fgf21 negatively regulates *Cyp7a1* expression (14) and Fgf21 is induced by PPARA activation (15, 16, 17, 18), we hypothesized that Fgf21 may play an important role in the effect of the PPARA agonist on inhibiting *Cyp7a1*. The effect of seladelpar was compared with that of a PPARA agonist. Gene expression of *Fgf21* in the liver (Fig. 4*A*) and serum Fgf21 (Fig. 4*B*) were significantly increased following gavage of seladelpar to WT C57BL/6 mice. Fgf21 concentration was higher in the supernatant of primary mouse hepatocytes stimulated with seladelpar (Fig. 4*C*). The gene expression of *Fgf21* in primary hepatocytes was significantly upregulated by seladelpar, the PPARA agonist Wy14643, and other PPARD agonists REN001 and ASP0367 (Figs. 4*D*, and S2*C*). To rule out the possibility that *Fgf21* induction by seladelpar was based on the activation of PPARA, primary hepatocytes isolated from *Ppara-*deficient mice were treated with seladelpar or Wy14643. As a result, only seladelpar induced the expression of *Fgf21* and *Pdk4* and inhibited *Cyp7a1* (Fig. 4, *G*–*I*). In addition, the effect of seladelpar was still observed in the presence of the selective PPARG antagonist GW9662 (Fig. S3). Furthermore, the effect of seladelpar on the expression of *Cyp7a1* and *Fgf21* was abolished by gene knockdown of *Ppard* (Fig. S4). These results indicate that seladelpar induces Fgf21 through PPARD activation.

### Fgf21 activates JNK signaling pathway to inhibit *Cyp7a1* expression

**Figure 5:**
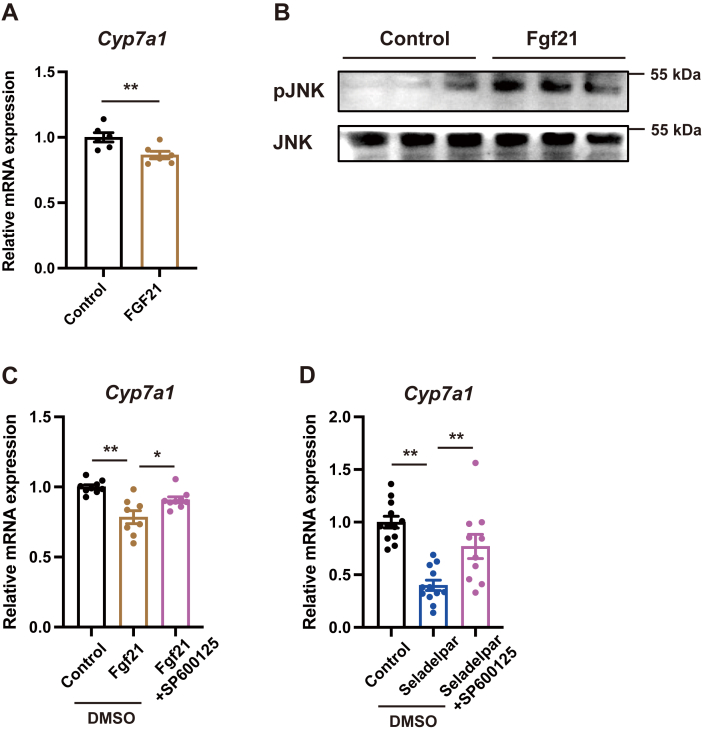
**Role of Fgf21 in the regulation of *Cyp7a1* expression in primary mouse hepatocytes.***A*, primary mouse hepatocytes were treated with recombinant Fgf21 protein (500 nM) for 4 h and mRNA expression of *Cyp7a1* was examined. *B*, primary mouse hepatocytes were treated with recombinant Fgf21 protein (500 nM) for 10 min, and immunoblots with anti-pJNK and anti-JNK antibodies were performed. *C*, primary mouse hepatocytes were treated with Fgf21 protein (500 nM) in combination with the JNK inhibitor SP600125 (40 μM) for 4 h. *D*, primary mouse hepatocytes were treated with seladelpar in combination with the JNK inhibitor SP600125 (40 μM) for 48 h. qPCR data are presented as mean ± S.E.M. of at least three independent replicates. ∗*p* < 0.05 and ∗∗*p* < 0.01 denote the significant difference between the groups. JNK, c-Jun N-terminal kinase.

Recombinant Fgf21 protein repressed the gene expression of *Cyp7a1* in primary mouse hepatocytes (Fig. 5*A*). In addition, Fgf21 activated the c-Jun N-terminal kinase (JNK) signaling pathway (Fig. 5*B*). The suppressive effect of Fgf21 on *Cyp7a1* expression was abolished by the JNK inhibitor SP600125 (Fig. 5*C*). Furthermore, SP600125 blocked the effect of seladelpar on *Cyp7a1* expression (Fig. 5*D*). Recombinant Fgf21 did not change the gene expression of *Ppard* or *Pdk4* (Fig. S5). These results indicate that PPARD-induced Fgf21 activates the JNK signaling pathway, which plays a role in reducing Cyp7a1 in hepatocytes.

### Seladelpar is unable to reduce Cyp7a1 in Fgf21-deficient mice

**Figure 6:**
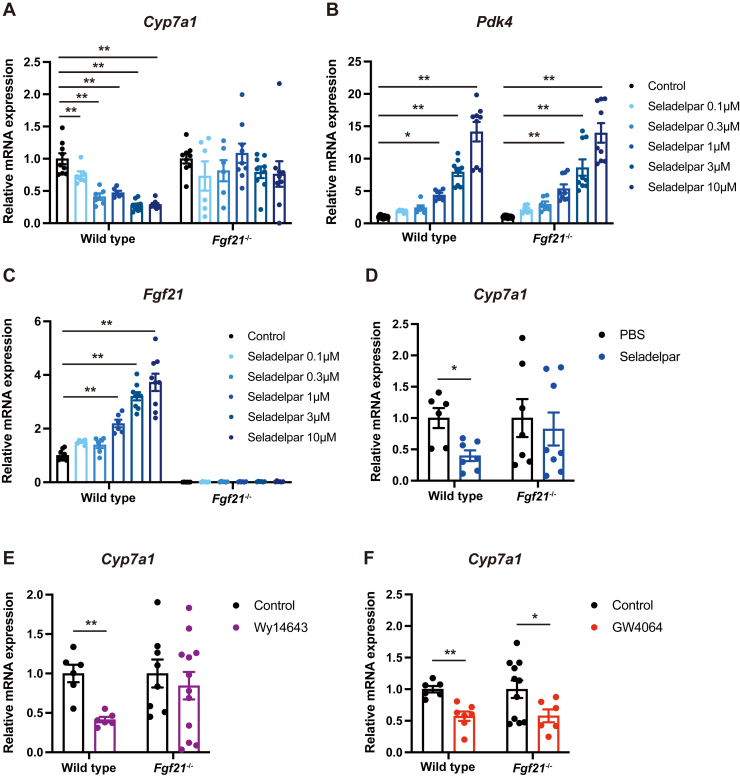
**Effect of seladelpar on *Cyp7a1* expression in *Fgf21* deficient hepatocytes.***A*–*C*, primary mouse hepatocytes isolated from WT or *Fgf21*^*-/-*^ were treated with seladelpar (0.1–10 μM). *E*, Wy14643 (10 μΜ) or *F*, GW4064 (10 μΜ) for 48 h. *D*, littermate WT and *Fgf21*^*-/-*^ were gavaged with PBS or seladelpar (10 mg/kg body weight) and harvested after 6 h. Hepatic expression of *Cyp7a1* was examined. qPCR data are presented as mean ± S.E.M. of at least three independent replicates. Two technical replicates were performed for mouse experiments. ∗*p* < 0.05 and ∗∗*p* < 0.01 denote the significant difference between control and treatment in each condition.

To determine the importance of Fgf21 in mediating the inhibitory effect of seladelpar on *Cyp7a1* expression, primary hepatocytes isolated from WT and *Fgf21*-deficient (*Fgf21*^*-/-*^) mice were treated with seladelpar, Wy14643, or GW4064. While seladelpar concentration dependently downregulated *Cyp7a1* gene expression in the WT hepatocytes, no significant change was observed in the cells isolated from *Fgf21*^*-/-*^ mice (Fig. 6*A*). *Pdk4* was induced by seladelpar in both cells (Fig. 6*B*), confirming PPARD activation. *Fgf21* was induced by seladelpar only in WT but not in *Fgf21*^*-/-*^ hepatocytes (Fig. 6*C*). Oral administration of seladelpar to WT mice significantly reduced hepatic expression of *Cyp7a1*, whereas no effect of seladelpar was observed in *Fgf21*^*-/-*^ mice (Fig. 6*D*). Similarly, the PPARA agonist Wy14643 suppressed the expression of *Cyp7a1* only in WT hepatocytes but not in *Fgf21*^*-/-*^ hepatocytes (Fig. 6*E*). The FXR agonist GW4064 reduced the expression of *Cyp7a1* in WT and *Fgf21*^*-/-*^ hepatocytes (Fig. 6*F*). These results indicate that Fgf21 plays an essential role in mediating the suppressive effect of seladelpar on *Cyp7a1* expression.

### Seladelpar reduces *CYP7A1* expression in primary human hepatocytes

**Figure 7:**
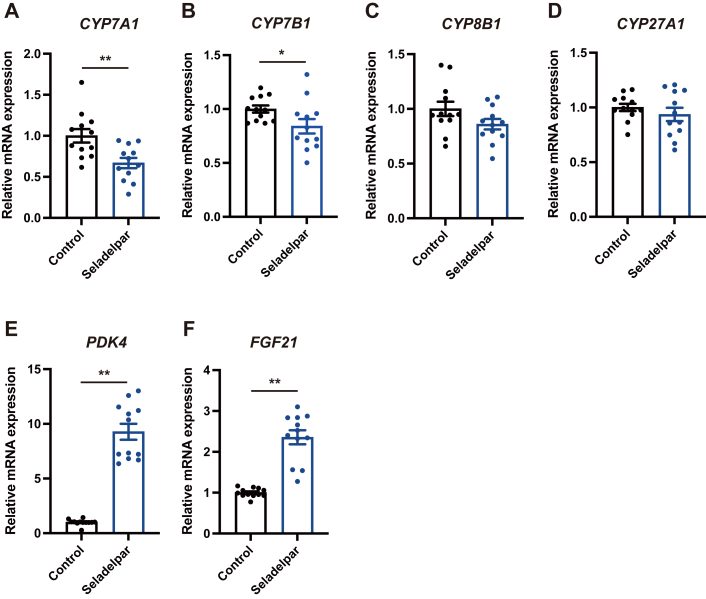
**Effect of seladelpar on *CYP7A1* expression in primary human hepatocytes.** Primary human hepatocytes were treated with seladelpar (10 μM) for 48 h and gene expression was analyzed by qPCR. Data are presented as mean ± S.E.M. of two independent replicates. ∗*p* < 0.05 and ∗∗*p* < 0.01 denote the significant difference between control and seladelpar.

To confirm our findings in human cells, primary human hepatocytes were treated with seladelpar. Seladelpar significantly reduced the gene expression of *CYP7A1* (Fig. 7*A*) and slightly downregulated *CYP7B1* (Fig. 7*B*), without affecting *CYP8B1* or *CYP27A1* (Fig. 7, *C* and *D*) and increased the expression of *PDK4* and *FGF21* (Fig. 7, *E* and *F*).

## Discussion

**Figure 8:**
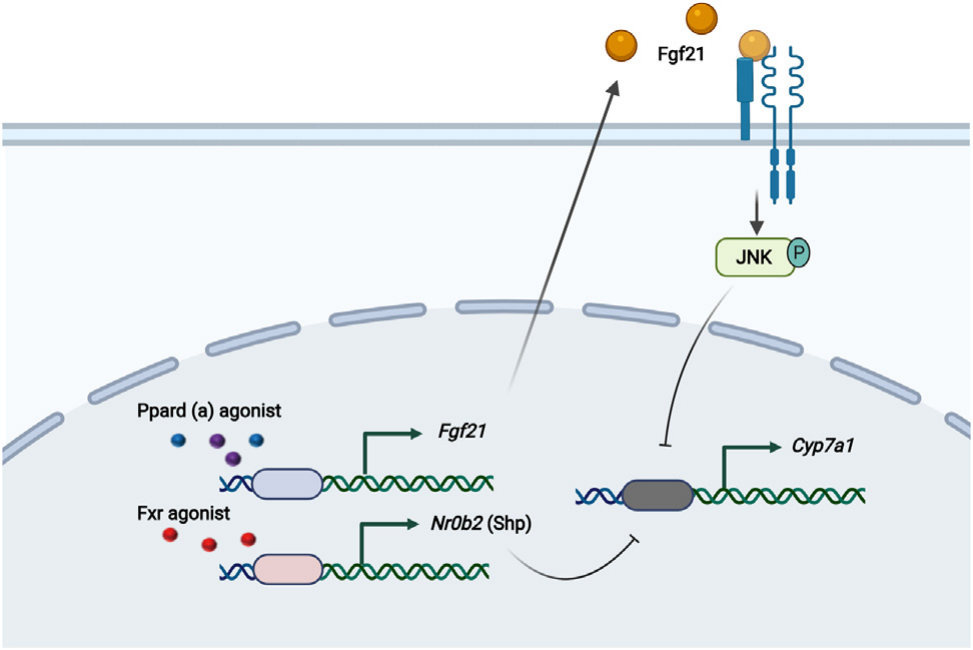
**Schematic diagram.** PPARD and PPARA agonists induce Fgf21 to activate JNK signaling pathway in hepatocytes, subsequently suppressing the transcription of Cyp7a1, which is independent from FXR/Shp pathway. Cartoon was created with BioRender.com . FXR, Farnesoid X receptor; JNK, c-Jun N-terminal kinase; PPARD, peroxisome proliferator–activated receptor delta; PPARA, peroxisome proliferator–activated receptor alpha; Shp, small heterodimer partner.

As a selective agonist of PPARD, seladelpar has demonstrated multiple beneficial effects in patients with either NASH (19) or PBC (12). Changes in bile acid composition and an increase in systemic bile acids are associated with both diseases (20, 21) and seladelpar decreased total bile acids in patients with PBC (12, 13). In humans, seladelpar administration decreases plasma C4 levels (12, 13, 19). Our present study is the first to describe that PPARD agonism decreases bile acid synthesis by repressing the expression of *Cyp7a1*, without affecting the alternative bile acid synthesis pathway and independent of the FXR pathway. We propose that seladelpar-mediated induction of Fgf21 plays an important role in reducing bile acid synthesis following PPARD agonism (Fig. 8).

Activation of PPARA strongly induces *Fgf21* in the liver (15, 16, 17, 18). By contrast, less is known about PPARD regulating hepatic Fgf21 expression. The PPARD receptor antagonist GSK0660 abolishes leptin-induced *Fgf21* expression in the adipose tissue of rats (22). In human subjects, plasma Fgf21 levels increase after the treatment with PPARD agonist GW590735 or PPARA agonist GW501516 (23). These studies indicate that PPARD activation induces hepatic expression of Fgf21. Contrary to these findings, *PPARD* deficiency results in increased serum and liver Fgf21 levels in mice (24). This discrepancy may be explained by an indirect mechanism of Fgf21 induction in *PPARD*-deficient mice. Two potential mechanisms can be postulated whereby Fgf21 is induced in PPARD KO mice. First, *PPARD* deficiency results in a reduction of peroxisome proliferator–activated receptor-gamma coactivator (Pgc)-1a expression and hemin levels, which, in turn, activates the heme-regulated eukaryotic translation initiation factor 2a (eIF2a) kinase. Phosphorylated elF2a increases activating transcription factor 4 (ATF4), which is responsible for inducing the hepatokine (25), resulting in enhanced expression of Fgf21. Second, PPARD binds B-cell lymphoma-6 (BCL-6), in an agonist independent manner, reducing BCL-6–mediated gene transcription. In *PPARD*^*-/-*^ animals or cells, BCL-6 is now available to maximize gene transcription (26, 27, 28). In our study, seladelpar induced *Fgf21* expression *in vivo* and in primary mouse and human hepatocytes. Importantly, hepatocytes isolated from *PPARA*^*-/-*^ mice, seladelpar still induced *Fgf21* and repressed *Cyp7a1*. Even though small differences in *Cyp7a1* expression between control and seladelpar were noted, seladelpar clearly induced the expression of *Fgf21* independent of PPARA.

Fgf21 has been shown to negatively regulate *Cyp7a1* expression in human hepatocytes and some rodent models (14). Consistent with this, we showed treatment with recombinant Fgf21 protein downregulated the gene expression of *Cyp7a1* in primary mouse hepatocytes. However, the effect of Fgf21 on bile acid homeostasis might vary depending on the experimental condition, since chronic overexpression of Fgf21 in mice increases the expression of *Cyp7a1* in the liver, resulting in an increased bile acid pool (29). Fgf21 shares the binding site to βKlotho with FGF15/19 (30, 31), and Fgf21 can antagonize the function of Fgf15-mediated inhibition of bile acid synthesis. Taking into account that Fgf15 more potently downregulates the expression of *Cyp7a1* than Fgf21 does in human hepatocytes (14), chronic overexpression of Fgf21 might work in favor of inhibiting the function of Fgf15 as a negative regulator of *Cyp7a1* expression in the latter study, resulting in an increase in *Cyp7a1* expression. On the other hand, a marker for *de novo* synthesis of bile acids and serum bile acids were decreased after 52 w of treatment with seladelpar in PBC patients (13), suggesting that transient induction of Fgf21 affects bile acid synthesis differently from chronic overexpression of Fgf21. Further studies are required to better understand the interaction between Fgf21- and Fgf15/19-mediated bile acid regulation.

JNK signaling regulates bile acid homeostasis independent of Shp in hepatocytes. For example, inflammatory cytokines, such as Interleukin 1 beta and tumor necrosis factor activate JNK signaling in hepatocytes to inhibit *Cyp7a1* expression (32, 33). Hepatic JNK deficiency alters bile acid homeostasis, causing cholestasis and liver damage (34). In our present study, Fgf21 activated JNK, while a JNK inhibitor blocked the suppressive effect of Fgf21 and seladelpar on *Cyp7a1* transcription, indicating that Fgf21 also regulates bile acid homeostasis through the JNK signaling pathway, and the effect of PPARD agonist on bile acid synthesis is at least partially mediated by the Fgf21/JNK cascade.

In summary, the selective PPARD agonist seladelpar reduces CYP7A1 in both mouse and human hepatocytes via induction of FGF21, not *via* PPARD repressive elements in the *CYP7A1* promotor. Hepatic expression of FGF21 is induced by PPARD agonism, and FGF21 plays a key, perhaps exclusive, role in the PPARD-mediated repression of CYP7A1 in the liver.

## Experimental procedures

### Reagents

Seladelpar (MBX-8025) was provided by CymaBay Therapeutics. Wy14643 (PPARα agonist) and GW4064 (FXR agonist) were purchased from MilliporeSigma. DY268 (FXR antagonist) was from Axon Medchem. PPARG antagonist GW9662 was from Tocris Bioscience. FGF21 recombinant protein was from Thermo Fisher Scientific (8409FG025). Anti-Cyp7a1 antibody was obtained from abcam (ab65595). Anti-GAPDH antibody was purchased from GeneTex (GTX100118, GeneTex). pJNK antibody (9251S) and SAPK/JNK antibody (9252S) were purchased from Cell Signaling. SP600125 (JNK inhibitor) was purchased from AdooQ BIOSCIENCE. ON-TARGET plus Mouse *Ppard* siRNAs and Non-targeting Control siRNA were purchased from Horizon Discovery.

### Mice

WT C57BL/6 mice were bred in the vivarium at UCSD. PPARα-deficient mice were purchased from The Jackson Laboratory. Fgf21-deficient mice and their WT littermate mice have been described (35). Male mice were gavaged with vehicle (PBS) or seladelpar (10 mg/kg body weight), and liver and ileum were harvested 6 h later. Mice had free access to food and water and were maintained on 12 h artificial light and dark cycle. All animal studies were reviewed and approved by the Institutional Animal Care and Use Committee of the University of California (UCSD).

### C4 measurement

C4 levels were determined using a tandem LC/MS/MS at Quintara Discovery as described (11).

### Isolation and culture of primary mouse hepatocytes

Primary mouse hepatocytes were isolated from 9- to 15-week-old mice. After mice were anesthetized using a ketamine/xylazine mixture, the vena cava was cannulated, and the liver was perfused for 5 min at 10 ml/min with perfusion buffer, followed by the buffer containing collagenase D and collagenase P (Roche Diagnostics) at 10 ml/min for 7 min. The liver was dissected from the mice and ruptured with forceps in Dulbecco’s modified Eagle’s medium (DMEM) containing 10% fetal bovine serum (FBS). The cells were filtered through a 70 μm cell strainer. Hepatocytes were obtained by centrifugation at 84*g* for 1 min. Isolated cells were plated in 6-well collagen-coated plates in DMEM containing 10% FBS, 0.35 μM insulin, and 0.1 μM dexamethasone. After a 3-h attachment, the cell medium was changed, and cells were treated with tested compounds in the medium with 10% FBS. Except for a dose response experiment in Figs. 2 and 6, a seladelpar concentration of 10 μM was used to treat isolated hepatocytes. This is similar to plasma concentrations in clinical trials (36).

### Isolation of primary human hepatocytes

Deidentified livers declined for transplantation were used in this study; the patient’s consent was obtained by www.lifesharing.org. This project (171883XX) has been reviewed by the Director of the UCSD HRPP, IRB Chair or the IRB Chair’s designee and is certified as not qualifying as human subjects research according to the Code of Federal Regulations, Title 45, Part 46 and UCSD Standard Operating Policies and Procedures and therefore does not require IRB review. Livers were graded for steatosis, inflammation, and fibrosis by a pathologist using a double-blinded method. Primary human hepatocytes were isolated as described (37).

### Real-time quantitative PCR

Total RNA was extracted from frozen tissue or hepatocytes using Trizol (Invitrogen). Complementary DNAs (cDNAs) were generated using the high-capacity cDNA reverse transcription kit (Applied Biosystems). The cDNA was amplified and quantified using SYBR Green (Bio-Rad Laboratories). Relative gene expression was determined by CT value and normalized to *Gapdh* as the housekeeping gene. Primer sequences are listed below:

Mouse *Gapdh* forward TTGATGGCAACAATCTCCAC

Mouse *Gapdh* reverse CGTCCCGTAGACAAAATGGT

Mouse *Cyp7a1* forward GGGAATGCCATTTACTTGGA

Mouse *Cyp7a1* reverse GTCCGGATATTCAAGGATGC

Mouse *Cyp7b1* forward TCCTAGGCCTTCTCTTTGCC

Mouse *Cyp7b1* reverse TTATCAAGGGTGGTTCACGA

Mouse *Cyp8b1* forward TCCTCAGGGTGGTACAGGAG

Mouse *Cyp8b1* reverse GATAGGGGAAGAGAGCCACC

Mouse *Cyp27a1* forward CTATGTGCTGCACTTGCCC

Mouse *Cyp27a1* reverse ACTTGCCCTCCTGTCTCATC

Mouse *Nr0b2* forward TCTGCAGGTCGTCCGACTATTC

Mouse *Nr0b2* reverse AGGCAGTGGCTGTGAGATGC

Mouse *Pdk4* forward GGGTCTCAATAGTGTCACC

Mouse *Pdk4* reverse GTGGGCCTGGGCATTTAGCA

Mouse *Angptl4* forward AAGATGACCCAGCTCATTGG

Mouse *Angptl4* reverse GGAAAAGTCCACTGTGCCTC

Mouse *Abcb11* forward AAGGACAGCCACACCAACTC

Mouse *Abcb11* reverse CCAGAACATGACAAACGGAA

Mouse *Fgf21* forward CCTGGGTGTCAAAGCCTCTA

Mouse *Fgf21* reverse CTCCAGCAGCAGTTCTCTGA

Mouse *Fgf15* forward GAGGACCAAAACGAACGAAATT

Mouse *Fgf15* reverse ACGTCCTTGATGGCAATCG

Mouse *Slc10a2* forward TGGTGTAGACGAAGAGGCAA

Mouse *Slc10a2* reverse GCCTATTGGATAGATGGCGA

Human *GAPDH* forward GTCTCCTCTGACTTCAACAGCG

Human *GAPDH* reverse ACCACCCTGTTGCTGTAGCCAA

Human *CYP7A1* forward GAGAAGGCAAACGGGTGAAC

Human *CYP7A1* reverse GGATTGGCACCAAATTGCAGA

Human *CYP7B1* forward GCTTCCTTATCTTGGAGTGG

Human *CYP7B1* reverse GAGCTGCAGAATGGATACAG

Human *CYP8B1* forward GCCGACTCCAGCGTCTCTC

Human *CYP8B1* reverse GCCCGCCGTTGCTGAGCT

Human *CYP27A1* forward TGCGCCAGGCTCTGAACCAG

Human *CYP27A1* reverse TCCACTTGGGGAGGAAGGTG

Human *PDK4* forward AGAGCCTGATGGATTTGGTG

Human *PDK4* reverse GCTTGGGTTTCCTGTCTGTG

Human *FGF21* forward CTGTGGGTTTCTGTGCTGG

Human *FGF21* reverse CCGGCTTCAAGGCTTTCAG

### Immunoblotting

Cell lysate was prepared with RIPA buffer (Thermo Fisher Scientific) containing protease inhibitors (Roche Diagnostics) and Halt phosphatase inhibitors (Thermo Fisher Scientific). Proteins were separated by SDS-PAGE and transferred to polyvinylidene difluoride membranes (Bio-Rad Laboratories). Immunoblot analysis was performed using antibodies dissolved in 5% bovine serum albumin in TBS containing 0.05% Tween 20. Protein bands were detected with ECL (Thermo Fisher Scientific) using an Imaging System (Bio-Rad Laboratories).

### ELISA

Mouse serum samples were prepared by centrifugation at 4000*g* for 20 min, and serum Fgf21 and Fgf15 were detected using the Mouse/rat Fgf21 Quantikine ELISA Kit (MF2100, R&D Systems) and the Mouse Fgf15 ELISA Kit (LS-F11446, LSBio) respectively, according to the manufacturer's instructions.

### siRNA mediated *Ppard* knockdown study

Three hours after the isolation, primary mouse hepatocytes were transfected with control siRNA or mouse *Ppard* siRNA using Lipofectamine 3000 Transfection Reagent (Invitrogen) according to the manufacturer’s instruction. After 24-h incubation, cells were treated with seladelpar for 48 h and then used for qPCR.

### Statistical analysis

All data are expressed as mean ± S.E.M. For comparison of two groups, an unpaired Student’s *t* test was performed. For multiple groups comparison within one experimental setting, one-way ANOVA with Dunnett’s post hoc test or Tukey’s post hoc test was conducted. Statistical analyses were performed with GraphPad Prism (V.9.2.0). A *p* value < 0.05 was considered significant.

## Data availability

All data are contained in the article.

## Supporting information

This article contains supporting information.

## Conflicts of interest

E. E. C. is employee of CymaBay. All other authors declare that they have no conflicts of interest with the contents of this article.
