# Supplementary material for: Selective PPARδ agonist seladelpar suppresses bile acid synthesis by reducing hepatocyte CYP7A1 via the fibroblast growth factor 21 signaling pathway
Source: J Biol Chem. 2022 May 20;298(7):102056. doi: 10.1016/j.jbc.2022.102056 (PMC9214809; doi:10.1016/j.jbc.2022.102056)
Supplement: Supporting Information Text [file mmc1.docx]

**Supporting Information**

**Figure legends**

**Figure S1. Effect of seladelpar on expression of *Ppar* genes**

(A-C) Gene expression in primary mouse hepatocytes treated with seladelpar (10 μM). Data are presented as mean±S.E.M. N.S., not significant.

**Figure S2. Effect of other PPARD agonists in primary mouse hepatocytes**

Primary mouse hepatocytes were treated with PPARD agonist REN001 (10 μM) or ASP0367 (30 μM) for 48 hours, and gene expression analysis was performed. Data are presented as mean±S.E.M. ***p*<0.01 denotes the significant difference between control and compounds.

**Figure S3. Effect of seladelpar in primary mouse hepatocytes treated with PPARG antagonist**

Primary mouse hepatocytes were treated with seladelpar (10 μM) in combination with PPARG antagonist GW9662 (10 μM) for 48 hours, and gene expression analysis was performed. Data are presented as mean±S.E.M. ***p*<0.01 denotes the significant difference.

**Figure S4. Effect of seladelpar in *Ppard* siRNA-treated primary mouse hepatocytes**

(A-C) Primary mouse hepatocytes were transfected with control or *Ppard* siRNA. 24 hours after the transfection, cells were treated with seladelpar (10 μM) for 48 hours, and gene expression analysis was performed. Data are presented as mean±S.E.M. **p*<0.05 and ***p*<0.01 denotes the significant difference between control and treated group.

**Figure S5. Effect of Fgf21 on Ppard function in primary mouse hepatocytes**

Primary mouse hepatocytes were treated with recombinant Fgf21 protein (500 nM) for 4 hours and gene expression analysis was performed. Data are presented as mean±S.E.M. N.S., not significant.
