# Supplementary figures and images for: Selective PPARδ agonist seladelpar suppresses bile acid synthesis by reducing hepatocyte CYP7A1 via the fibroblast growth factor 21 signaling pathway
Source: J Biol Chem. 2022 May 20;298(7):102056. doi: 10.1016/j.jbc.2022.102056 (PMC9214809; doi:10.1016/j.jbc.2022.102056)

Figure S1

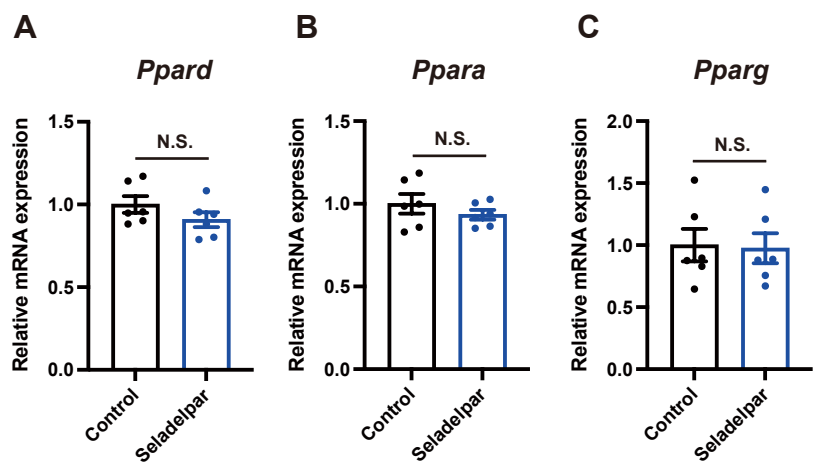

Figure S2

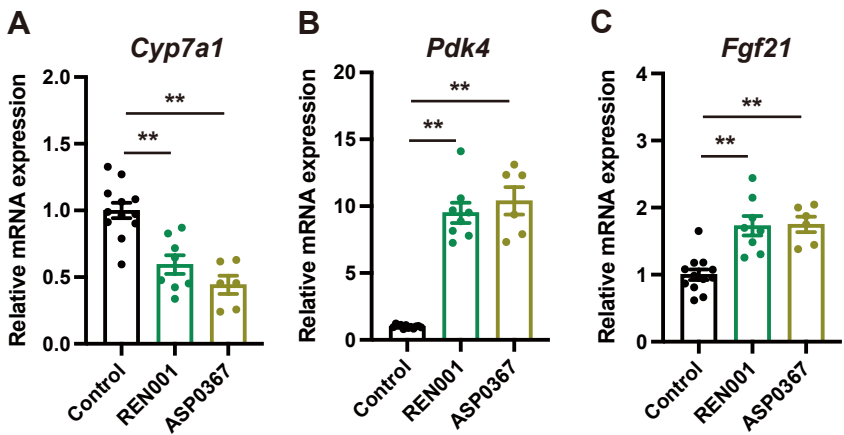

Figure S3

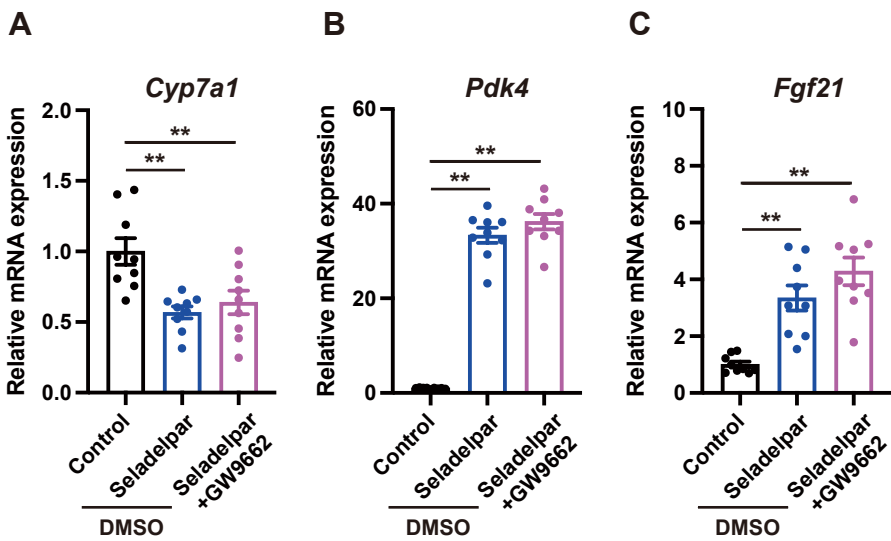

Figure S4

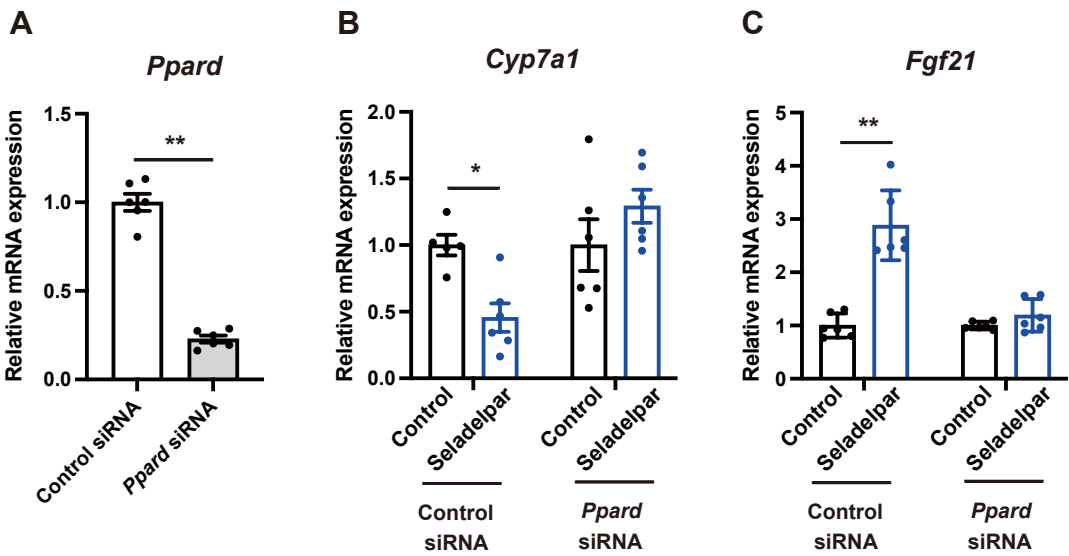

Figure S5

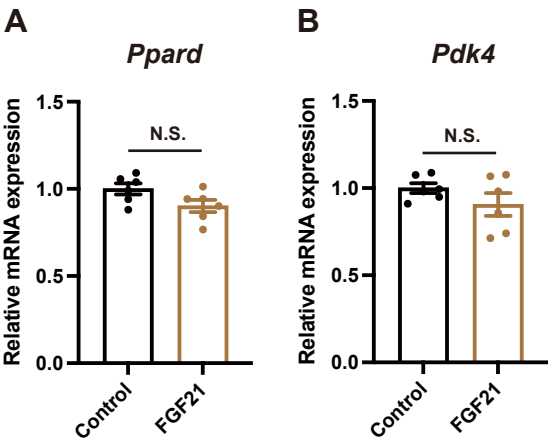

Supplement: Supporting Information Figures [file mmc2.pdf]
